# HPLC separation and *in vitro* antimalarial studies of *Artemisia annua* plants from two different origins: Cameroon versus Luxembourg

**DOI:** 10.5281/zenodo.10887904

**Published:** 2014-11-16

**Authors:** Mutaz Akkawi, Suhair Jaber, Saleh Abu-Lafi, Mutaz Qutob, Qassem Abu-Rmeleh, Pierre Lutgen

**Affiliations:** 1 Life Sciences Department, College of Science and Technology, Al-Quds University, West Bank, Palestine; 2 Faculty of Pharmacy, Al-Quds University, West Bank, Palestine; 3 Department of Earth and Environmental Sciences, College of Science and Technology, Al-Quds University, West Bank, Palestine; 4 IFBV-BELHERB, Luxembourg

## Abstract

**Background:**

Malaria is a devastating disease, particularly in Africa, due to development of resistance by *Plasmodium falciparum* against all known antimalarial drugs, including artemisinin. Therefore, the search for new antimalarial drugs is urgently needed, especially drugs that can impede the heme detoxification pathway in the malaria parasite, a crucial requirement for parasite survival in host erythrocytes.

**Materials and Methods:**

Water infusions of *Artemisia annua* plants from two different origins, Cameroon and Luxembourg, were used in this study. A semi-quantitative *in vitro* method, based on the inhibition of ferriprotoporphyrin IX (FP) biomineralisation developed by Deharo *et al.* [16], was used to reveal the differences in antimalarial activity of both plants. Reversed phase preparative liquid chromatography coupled to a photo diode array (PDA) detector was also used to test for differences in antimalarial activity.

**Results:**

Water extracts from the leaves of the Cameroon plant showed a higher potential antimalarial activity, represented by a higher ability to inhibit *β*-haematin formation *in vitro* than *A. annua* extracts from Luxembourg. Although extracts of the plants of both origins showed comparable efficiencies at high concentrations, the absorbance value at 405 nm of a 10% dilution of the Cameroon plant extract was 0.075, whereas it was 1.515 for the Luxembourg plant extract. The absorbance is inversely proportional to the antimalarial activity. According to the Prep-HPLC chromatogram of the Cameroon crude sample, seven major compounds at 325 nm were found. However, only four much less pronounced compounds appeared in the Luxembourg crude sample under the same chromatographic conditions and concentration. These were preliminarily identified as polyphenolic compounds.

**Conclusion:**

*A. annua* infusions are widely used by people who cannot afford other treatments. Depending on the cultivation locality different chemical profiles exist. This results in differences in hemozoin formation and will therefore also lead to alterations in antimalarial activity.

## 1 Introduction

Malaria remains a serious global health problem, especially in developing countries, where children under the age of five and pregnant women are especially at risk [1]. The most important cause for this alarming situation is the rapid spread of malaria parasites that are resistant to antimalarial drugs currently used, including artemisinin. Human malaria is caused by one of four protozoa, *Plasmodium malariae*, *P*. *ovale*, *P*. *vivax* and *P*. *falciparum*; the latter being the most important cause of morbidity and mortality [2].

During the intra-erythrocytic stage of the parasite life cycle, the growing parasite consumes the intercellular proteins in red blood cells (RBCs), mainly haemoglobin, as a source of amino acids. Digestion of haemoglobin takes place inside the parasite food vacuoles, where the pH range is between 5.0 and 5.4 [3]. Massive degradation of haemoglobin leads to the generation of large amounts of free oxidized heme, which is extremely toxic to the parasite, leading to damage of the parasite membrane due to oxidative stress. However, the malaria parasite has developed a unique mechanism for heme detoxification through its bio-mineralisation into an insoluble, inert, crystalline, black-brown pigment named hemozoin [4]. This prevents the accumulation of toxic free heme, thus making this process crucial for parasite survival. Inhibition of hemozoin formation is considered to be the most suitable target to develop new anti-malarials.

Hemozoin is a polymer made of dimers of haematin molecules that are connected by hydrogen bonds. These dimers are formed through an iron-oxygen coordinate bond that links the central ferric iron of one haematin to the carboxylate side group oxygen of another haematin [3-7]. Several antimalarial drugs, such as chloroquine [3,5], are thought to exert their effect by complexing with free heme in the food vacuole, thus inhibiting its detoxification.

A crystalline synthetic structure known as *β*-haematin, having the same linkage between the heme groups, is believed to be structurally, morphology and spectroscopically identical to purified hemozoin [6]. *β*-haematin formation could be accomplished *in vitro* under specific chemical conditions (acidic pH) through a biocrystallisation process, thus making it an outstanding target for *in vitro* screening of antimalarial compounds [8,9].

Natural products from medicinal plants continue to have an essential function in medicine and provide natural polytherapies. Most of the world’s population, mainly in developing countries, relies on natural products for medication. Due to a renewed interest and the growing use of medicinal plants, it is of great importance to study commonly used medicinal plants in detail. The genus *Artemisia*, belonging to the Asteraceae family, is diverse and consists of about 400 species. Some very important drugs have been discovered from this genus, especially artemisinin, a secondary metabolite isolated from the Chinese herb *A. annua.* It was identified as a sesquiterpene lactone endoperoxide [10] and its derivatives have become the main weapon in the fight against malaria [11].

In continuation of our previous research on medicinal plants with antimalarial activity [12-15], we conducted a series of quantitative HPLC separations and studied the effect on hemozoin formation in order to explain the differences in antimalarial activity of *A. annua* plants from two different origins: Cameroon and Luxembourg.

## 2 Materials and Methods

HPLC-grade glacial acetic acid, acetonitrile (ACN) and ethanol (EtOH) were all purchased from Merck (Germany). Dimethyl sulfoxide (DMSO), purity 99.5%, chloroquine diphosphate salt, sodium acetate, purity 99%, and haemin chloride were all obtained from Sigma-Aldrich.

### 2.1 Collection and extraction

Samples of *A. annua* leaves were collected from Luxembourg in 2012 and from Cameroon in 2009. The sample from Luxembourg was of the arteannuin-B type and the sample from Cameroon of the artemisinin type. Both plants were harvested at the early stages of flowering. Both cultivates were grown without inorganic fertilizer.

Infusions were obtained by soaking 2 g of the plant material in 150 ml of distilled hot water at 90°C, left for 20 minutes at room temperature, then filtered using MN 615 Ø110 mm filter paper. Dried, concentrated extracts from the infusions were obtained by evaporation of the water at 60-70°C under reduced pressure using a rotary evaporator (IKA WEREKRV06-ML), followed by freeze-drying (Labconco freeze drier) until a constant weight was achieved. The final dried extract was stored in opaque bottles and kept in desiccators until analysis by preparative HPLC.

### 2.2 *In vitro* semi-quantitative test for screening of anti-malarial activity

Following the protocol by Deharo et al. [16], a mixture containing 50 μl of 0.5 mg/ml haemin chloride (freshly dissolved in DMSO), 100 μl of 0.5 M sodium acetate buffer (pH 4.4) and 50 μl of the tested anti-malarial drug solution or control, was incubated in a normal non-sterile 96-well flat bottom plate at 37ºC for 18-24 hours. It is imperative that the solutions be added to the plate in this order. The plate was then centrifuged for 10 minutes at 4000 rpm. The supernatant was removed and the pH of reaction was measured. The final pH of the mixture should be between 5.0 and 5.2. The wells were washed with 200 μl DMSO per well to remove free haemin chloride. The plate was centrifuged again, discharging the supernatant afterwards. The *β*-haematin remaining was then dissolved in 200 μl of 0.1 M NaOH to form an FP that could be measured spectrophotometrically. Finally, the absorbance was determined at 405 nm using an ELISA reader (Stat Fax-2100). Ultrapure water was used as negative control, whereas chloroquine dissolved in ultrapure water was used as positive control.

### 2.3 Chromatographic analysis

The Analytical High Pressure Liquid Chromatography (HPLC-PDA) system consists of an alliance 2695 HPLC, 2996-Photo Diode Array (PDA). Data acquisition and control were carried out using Empower software (Waters, Germany). The Preparative High Pressure Liquid Chromatography (Prep-HPLC-PDA) system consisted of a 3535 quaternary gradient module, which provides a flow rate of up to 45 ml/min, equipped with 996 PDA detectors (Waters, Germany).

The mobile phase was prepared by mixing acidic ultra-purified water (containing 0.05% glacial acetic acid) with acetonitrile (ACN) in the gradient mode. The acidic water phase was filtered by using a 0.45 mm microporous filter and was degassed by sonication prior to use. The HPLC analytical experiments were run on an octadecylsilane C18 chemically bonded column (Waters XBridge, 4.6 x 150 mm, 5 μm). The flow rate was 1 ml/min. The injection volume was 10 ml of 2mg/ml of the dried extract. Analyses were performed at room temperature. The mobile phase consisted of (A) 0.05% acetic acid in water and (B) ACN. The applied linear gradient was 90-0% A in 25 min. Before analysis, the column was equilibrated with the starting mixture for 10 minutes. A wavelength of 325 nm was chosen as it was found to be the most appropriate for the determination of all major separated peaks.

The HPLC Preparative experiments were run on ODS column (Agilent PrepHT C18, 22.2 x 250 mm, 10 μm). The same mobile phase and gradient were used. The applied flow rate used was 20 ml/min and the injection volume was 1000 ml. Total run time of last eluting compound was about 10 minutes.

Sample solutions were prepared by dissolving 50 mg of dry extract in 5 ml acidic water (0.05%), first by mechanical shaking for 3 min, followed by sonification for 1 min. The obtained solution was filtered using a 0.45 mm membrane filter (PVDF) before injection into the Prep-HPLC.

## 3 Results

### 3.1 Influence on hemozoin formation

**Figure 1.**
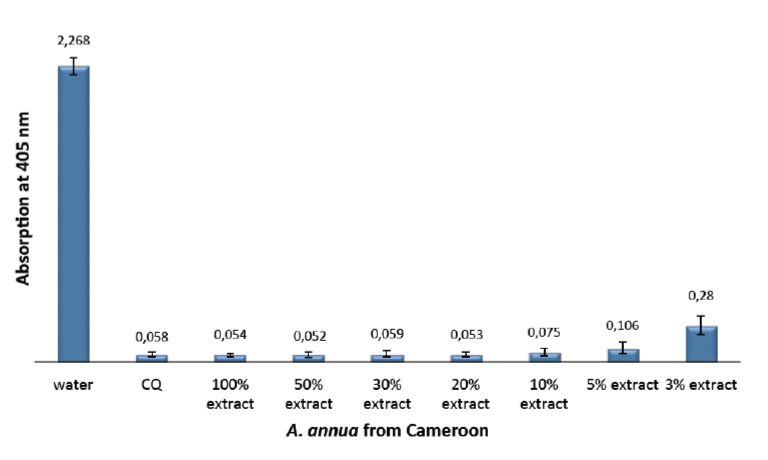
Efficiency of *A. annua* infusion from Cameroon compared with the negative (water) and positive control (CQ-chloroquine 0.1 mg/ml), showing the absorption values of dissolved *β*-haematin (alkaline haematin) at 405 nm. The absorption is inversely proportional to compound efficiency. Each result represents the average of 16 individual experiments.

**Figure 2.**
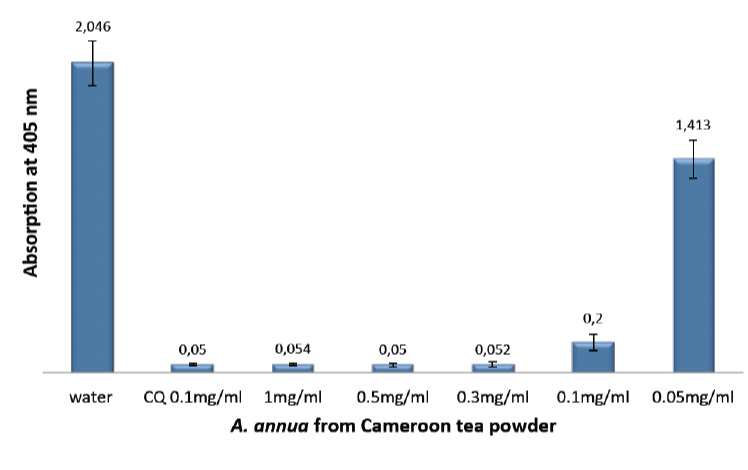
Efficiency of *A. annua* lyophilised infusion from Cameroon, compared with the negative (water) and positive control (CQ-chloroquine 0.1 mg/ml), showing the absorption values of dissolved *β*-haematin (alkaline haematin) at 405 nm. The absorption is inversely proportional to compound efficiency. Each result represents the average of 16 individual experiments.

Fig. 1 shows the inhibitory effect on hemozoin formation of different dilutions of *A. annua* tea infusion obtained on base of the Cameroon sample, while in Fig. 2 the test results after lyophilisation of the hot water tea infusion of the plant are expressed. According to the semi-quantitative method used in this research, the absorption is inversely proportional to the hemozoin content. The lower the absorption, the stronger the inhibitory effect towards hemazoin formation.

These results can be compared with previously published results [14] that had been obtained by using the same method and same plant species, but from a different origin (Luxembourg). Here, the potential antimalarial activity, represented by the ability to inhibit *β*-haematin formation, was lower compared with the Cameroon plant extracts. The absorbance at 405 nm of a 10% dilution of the Luxembourg plant was 1.515, whereas a value of 0.075 was obtained for the same dilution of the Cameroon plant extract. This strong difference in activity may be attributed to differences in the amount of polyphenols in each plant sample and to the ability of these chemicals to form non-covalent interactions (hydrogen bonding) with ferriheme. Table 1 summarises the results on *β*-haematin inhibitory effect of different dilutions of tea infusion from *A. annua* leaves obtained from the two different origins (Cameroon and Luxembourg). In addition, the effects measured with two other *Artemisia* species, *A. afra* and *A. sieberi*, are included.

**Table 1.** Efficiency of infusions of different *Artemisia* species, showing the absorption values of dissolved *β*-haematin (alkaline haematin) at 405 nm. Each result represents the average of 16 individual experiments.

**Species and origin**	**Absorption values at 405 nm for different dilutions**			
	100%	30%	10%	5%
*A. annua* Cameroon	0.054	0.059	0.075	0.106
*A. annua* Luxembourg	0.098	0.158	1.515	eq
*A. afra*	0.125	0.109	0.263	0.600
*A. sieberi*	0.106	0.142	1.732	eq

eq: no significant difference compared with pure water

### 3.2 Chromatographic analysis

**Figure 3.**
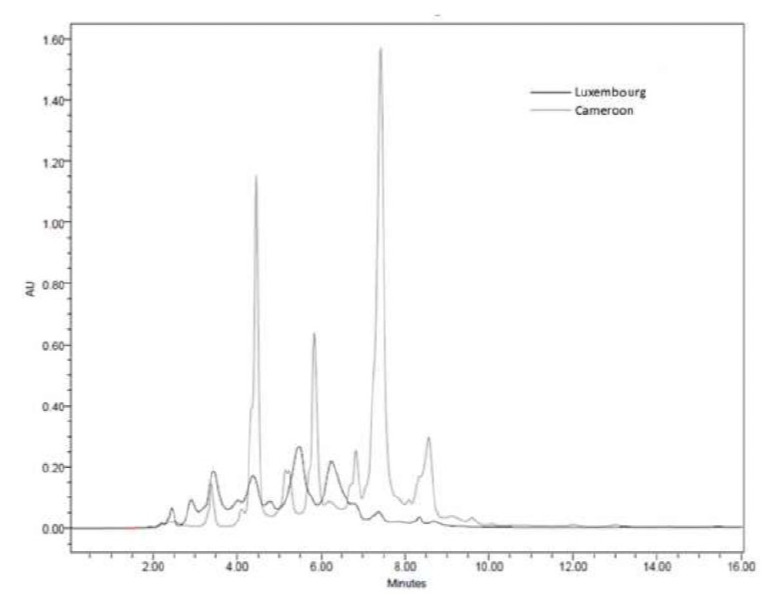
Overlay of preparative HPLC chromatograms of Cameroon and Luxembourg crude mixtures.

**Figure 4.**
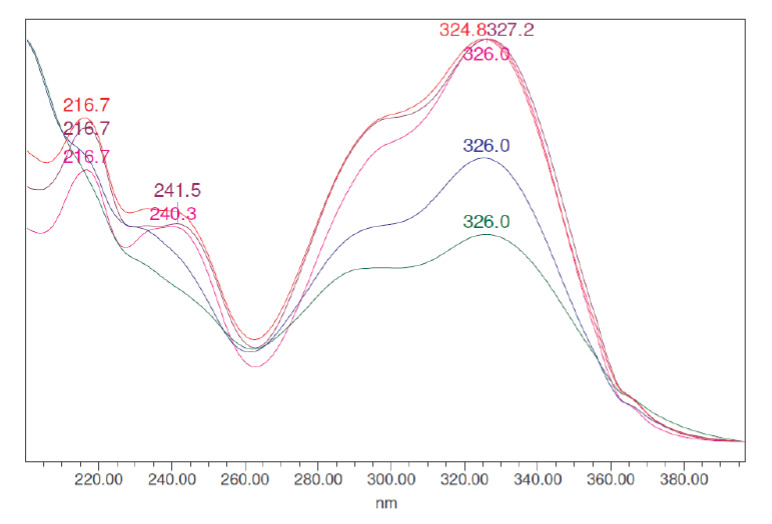
Overlay of UV-visible spectra of the major eluted peaks from the Cameroon crude mixture.

An overlay of the results obtained with preparative HPLC of the concentrated extracts is shown in Fig. 3. One ml of this solution was directly injected into the preparative HPLC (10 mg each). Comparison of the Prep-HPLC compositional profiles disclosed a significant difference between the two selected extracts using the same concentration and experimental conditions accounting for their biological differences. The overlay of the UV-visible spectra of the major peaks obtained with the concentrated extract from the Cameroon sample mixture is shown in Fig. 4. Chromatogram differences of the extracts by the photodiode array detector revealed that in addition to compounds in common, other metabolites were present in the water extracts sharing quite similar UV-Vis spectral absorbance.

## 4 Discussion

When comparing the *in vitro* activity of *A. annua* plant extracts of both origins, a higher activity of the Cameroon plant was observed for all concentrations tested. The soils of the two locations have been analysed by the Laboratoire de l’Institut Technique Agricole of Luxembourg and do not show striking differences or deficiencies in essential minerals.

Conventionally, *A. annua* tea is considered a rich source of antioxidant phenolics (mainly flavonoids) and artemisinin [17,18] and it has been suggested that flavonoids and other substances present in the tea might improve the efficacy of artemisinin [19].

In a previous study by Lehane *et al.* [20], 11 purified flavonoids from dietary sources were tested for their antimalarial activity *in vitro* against chloroquine-sensitive and chloroquine-resistant strains. The most effective flavonoids were shown to be luteolin and quercetin, followed by apigenin.

*A. annua* cultivated in Cameroon has a different chemical profile than the same plant being cultivated in Luxembourg. This might be due to differing climatic conditions and other environmental influences.

A typical Prep-HPLC chromatogram of the Cameroon concentrated sample revealed seven major compounds at 325 nm (Fig. 3). However, four major, less pronounced, compounds appeared in the Luxembourg concentrated sample under the same chromatographic conditions and concentration. Only two major peaks share the same retention time in both samples, namely at 3.42 and 4.45 minutes, respectively. They also showed the same UV-visible spectra (Fig. 4). For some smaller peaks in the Luxembourg sample, a match in retention time was also found on 7.0, 7.5 and 8.5 min. On 6.25 min, a match for a smaller peak of the Cameroon sample was observed.

The UV-visible spectra of the peaks from the Cameroon sample concentrated extract indicate that these peaks may correspond with polyphenolic compounds (Fig. 4). These compounds are rich in conjugated bounds and therefore show an absorption maximum at 325 nm. According to Fig. 3, the total amounts of compounds present were much higher in the Cameroon sample than in the Luxembourg sample. It is striking that this also corresponds with an improved activity towards hemozoin formation observed for the Cameroon sample. Over the years, several laboratories have made a comparative analysis of the Cameroon and Luxembourg *A. annua* samples.

A UPLC analysis run at the Celabor laboratory in 2010 (P. Lutgen, *pers. comm.*) showed that a plant from Cameroon contained twice as many flavonoids and polyphenols. It contained ten times more artemisinin (1.2%), three times as much scopoletin and four times more rutin. The sample from Luxembourg was, however, four times richer in chlorogenic acid. A qualitative froth test for saponins has been performed by P. Lutgen on samples from Luxembourg and Cameroon. The latter samples are void of saponins, while those from Luxembourg yielded a strong froth. An extensive analysis of the essential oil present in *A. an-nua* samples from Cameroon and Luxembourg was also run by R. C. Kengwe at the Laboratoire National de la Santé in Luxembourg in 2010. Although differences were found, they were not large enough to elaborate a working hypothesis.

## 5 Conclusions

Currently, there is an urgent need for antimalarial drugs due to the increasing spread of resistant strains. *A. annua* is a medicinal plant used in traditional Chinese medicine as herbal tea to treat malaria. *A. annua* plantations are widespread and remain the only hope for many people who cannot afford other treatments. *A. annua* cultivated in Cameroon by small farmers may have a different chemical profile than plants cultivated in Luxembourg. This might be due to different genotypes of *A. annua*. Differences in climatic conditions and other environmental influences might also affect the inhibition efficiency. This probably leads to a higher antimalarial efficiency, as indicated by our results on the hemozoin inhibitory effect. Differences in the inhibitory effect of other *Artemisia* species on *β*-haematin have also been observed, and HPLC analysis on these *Artemisia* species are planned by our group.
